# Management einer chronischen vorderen, verhakten Schulterluxation während des COVID-19-Lockdowns

**DOI:** 10.1007/s00142-021-00449-8

**Published:** 2021-02-25

**Authors:** Oliver Tenfelde, Sebastian Imach, Paola Kappel, Arasch Wafaisade

**Affiliations:** Lehrstuhl für Unfallchirurgie, Orthopädie und Sporttraumatologie, Klinikum Köln-Merheim, Universität Witten/Herdecke, Ostmerheimer Str. 200, 51109 Köln, Deutschland

**Keywords:** Glenoidfraktur, Glenoidaugmentation, Tuberculum-minus-Osteotomie, Hill-Sachs-Läsion, Offene Reposition, Glenoid fracture, Glenoid augmentation, Lesser tuberosity osteotomy, Hill-Sachs lesion, Open reduction

## Abstract

In diesem Beitrag wird der Fall einer chronisch verhakten vorderen Schulterluxation bei einem 25-jährigen, geistig retardierten Patienten geschildert, welcher während des ersten COVID-19-Lockdowns eine verspätete orthopädisch-fachärztliche Versorgung erhielt. Die Therapie bestand in der offenen Reposition mittels Tuberculum-minus-Osteotomie, einer Auffüllung des Hill-Sachs-Defekts sowie einer knöchernen Glenoidaugmentation, jeweils mit autologem trikortikalem Beckenkammspan. Die hierzulande seltene Verletzung zeigt in Ländern mit erschwertem Zugang zum Gesundheitssystem eine höhere Inzidenz mit hochgradiger Funktionseinschränkung.

## Abstract (en)

This article describes the case of a chronically dislocated anterior shoulder in a 25-year-old mentally retarded patient, who received delayed orthopedic specialist care during the first COVID-19 lockdown. The therapy consisted of open reduction using lesser tuberosity osteotomy, filling of the Hill-Sachs defect and anterior glenoid augmentation, each with autologous tricortical bone graft of the iliac crest. While this injury is rare in countries with an advanced healthcare system, studies from countries with limited access to healthcare show a higher incidence, often with higher-grade functional impairment.

## Anamnese

Ein 25-jähriger Patient wurde in Begleitung seiner Mutter in der Notaufnahme vorstellig. Er berichtete von seit 3 Monaten bestehenden Schmerzen der rechten Schulter. Ein genaues Unfallereignis ließ sich auf Grund einer erschwerten Anamnese bei geistiger Retardierung des Patienten durch hypoxischen Hirnschaden nicht rekonstruieren. Durch das im April 2020 herrschende Besuchsverbot im Rahmen des COVID-19-Lockdowns sei es der Mutter nicht möglich gewesen, den in einem Heim untergebrachten Sohn zu besuchen. Am Telefon hatte der körperlich sehr aktive Patient immer wieder über Schulterschmerzen geklagt. Der hinzugezogene Hausarzt hätte eine lokale sowie systemische Analgesie verordnet. Schließlich erfolgte auf Eigeninitiative der Mutter die Vorstellung beim niedergelassenen Orthopäden, welcher den Patienten nach Röntgendiagnostik mit vorderer Schulterluxation in das Krankenhaus einwies.

## Klinischer Befund

Inspektorisch zeigte sich eine abgeflachte Kontur des M. deltoideus mit hervorstehendem Akromion. Eine aktive Bewegung war bis 50° Abduktion und Anteversion möglich. Der Haut- und Weichteilmantel waren intakt, es bestanden keine Prellmarken. Die periphere Durchblutung, Motorik und Sensibilität waren, soweit beurteilbar, intakt. Palpatorisch war eine leere Gelenkpfanne zu ertasten. Der initiale Constant-Murley-Score betrug 45 Punkte.

## Radiologischer Befund

**Figure 1:**
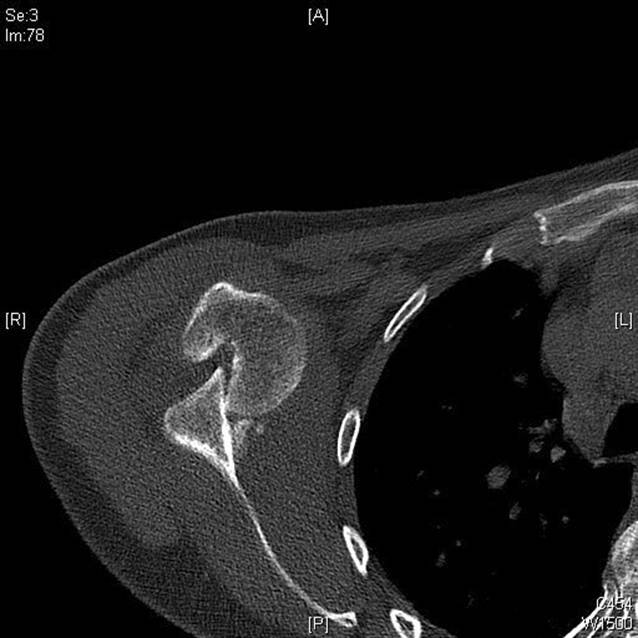

Auf der mitgeführten Röntgenaufnahme der Schulter in 2 Ebenen sowie der folgenden Computertomographie (CT) zeigte sich eine vordere Schulterluxation mit einem tiefgreifenden, V‑förmigen dorsalen Defekt des Humeruskopfes im Sinne einer ausgeprägten Hill-Sachs-Delle. Diese umfasste in der Tiefe etwa die Hälfte des Humeruskopfdurchmessers. Ebenso zeigte sich ein medial disloziertes, residuelles knöchernes Fragment des ventralen Glenoids im Sinne einer knöchernen Bankart-Läsion (Abb. 1).

## Therapie

**Figure 2:**
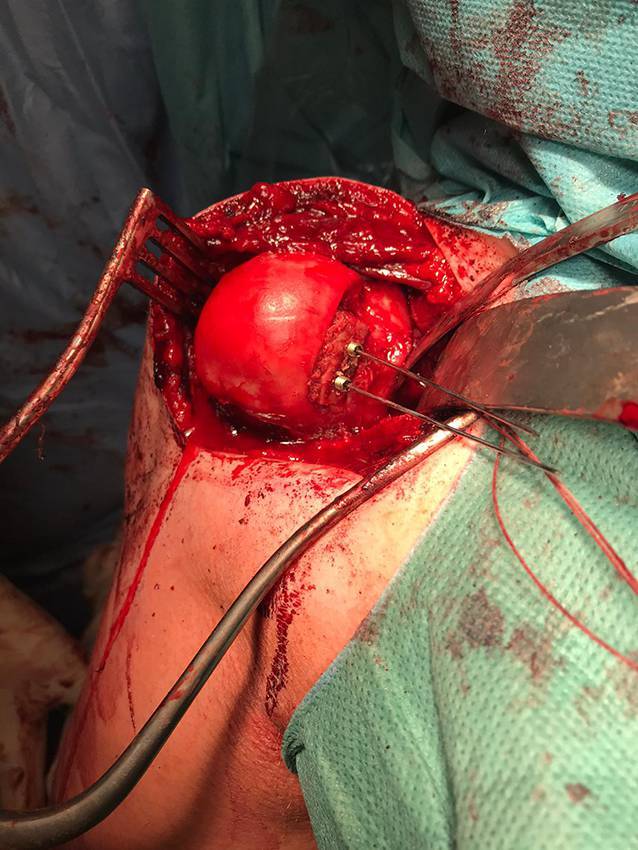

Der Eingriff wurde in Beach-chair-Lagerung in Allgemeinanästhesie durchgeführt. Es erfolgte zunächst die offene Darstellung über einen deltoideopektoralen Zugang. Nach Identifikation und Tenotomie der langen Bizepssehne wurde das Tuberculum minus dargestellt und mit dem Meißel osteotomiert. Danach gelang unter forcierter Außenrotation die Reposition. Der ventrale Skapulahals war arrodiert und der ventrale Labrumkomplex nicht mehr abgrenzbar. In maximaler Außenrotation zeigte sich ein tiefer, V‑förmiger Hill-Sachs-Defekt. Es wurde in typischer Technik ein trikortikaler Beckenkammspan entnommen und nach Anfrischen in den Hill-Sachs-Defekt eingepasst (Abb. 2). Der Beckenkammspan wurde zunächst mit 2 K-Drähten fixiert. Darüber wurden 2 versenkbare Doppelgewindeschrauben (APTUS®, Fa. Medartis [Basel, Schweiz], 2,2 mm) eingebracht. Ein zweiter Knochenspan wurde mit dem Zielinstrumentarium nach Taverna (Fa. Smith&Nephew [London, UK]) positioniert [9], mit kanülierten K‑Drähten fixiert und schließlich mit zwei entsprechenden Endobutton-Konstrukten fixiert. Abschließend wurde das Tuberculum minus unter Bildwandlerkontrolle mit 2 kanülierten Schrauben und entsprechenden Unterlegscheiben (ASNIS 4,0 mm, Fa. Stryker [Kalamazoo, MI, USA]) refixiert.

Die Nachbehandlung beinhaltete das Tragen einer Schulterorthese mit passiver krankengymnastischer Beübung des Schultergelenks für 6 Wochen mit zunehmender Steigerung des Bewegungsumfangs. Ab der 6. Woche durfte mit der aktiven Bewegung begonnen werden.

**Figure 3:**
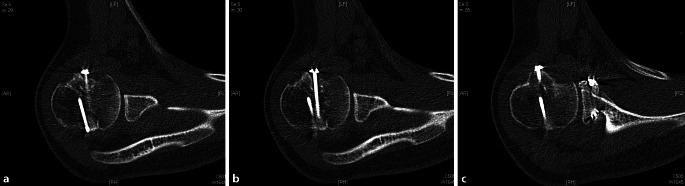

Bereits beim ersten Follow-up, 3 Monate postoperativ, zeigte sich eine stabile Gelenkführung und schmerzfreie Beweglichkeit, bei allerdings noch bestehender Bewegungseinschränkung mit einem Constant-Murley-Score von 59 Punkten. In der CT zeigten sich alle Anteile knöchern konsolidiert (Abb. 3).

## Diskussion

Die anteriore Schulterluxation ist eine häufige Verletzung, die im Rahmen der Notfallversorgung mittels geschlossener Reposition adressiert wird. Im Verlauf ist dann ggf. eine operative Behandlung von destabilisierenden Verletzungen erforderlich. In seltenen Fällen bleibt trotz Luxationsstellung des Gelenks eine gute Funktion erhalten, was zu einem Übersehen der Verletzung führen kann. Bleibt eine solche Verletzung für eine längere Zeit unerkannt, spricht man von einer chronischen oder verhakten Schulterluxation. Während die meisten Autoren eine chronische Schulterluxation als eine über mehr als 3 Wochen bestehende Luxationsdauer definieren, sind in der Literatur Angaben zwischen 24 h [7] und 1 Monat zu finden. Eine einheitliche Nomenklatur existiert nicht. Die Einteilung nach Goga et al. [5] gliedert die Verletzung in eine frühe (1–3 Wochen), eine späte (3–12 Wochen) und eine alte chronische Luxation (über 12 Wochen).

Je länger das Gelenk disloziert steht, desto schwieriger ist eine geschlossene Reposition

Je länger das Gelenk disloziert steht, desto schwieriger ist eine geschlossene Reposition. Häufig muss auf Grund der rasch eintretenden Fibrose eine offene Reposition durchgeführt werden. In Ländern mit eingeschränkter Gesundheitsversorgung tritt diese Verletzung häufiger auf. Eine südafrikanische Studie von Grey et al. [6] schloss retrospektiv 26 Patienten ein. Die durchschnittliche Luxationsdauer betrug 9 Monate. Gründe für die Chronifizierung waren eine verspätete Vorstellung beim Arzt, ein Übersehen der Verletzung durch den Arzt, aber auch eine mangelhafte Behandlung. Eine Hill-Sachs-Läsion bestand in 83 % der Fälle, eine geschlossene Reposition war in keinem der eingeschlossenen Fälle möglich.

In der Literatur existiert keine evidenzbasierte Handlungsempfehlung. Studien mit kleinen Fallzahlen zeigen jedoch gute Ergebnisse nach operativer Therapie [1, 2], wobei die konservative Therapie mit schlechten Langzeitergebnissen einhergeht [1, 3]. Bei der operativen Therapie steht sowohl die ventrale Schulterstabilisierung als auch die Behandlung der knöchernen Begleitverletzungen im Fokus. Die knöcherne, ventrale Schulterstabilisierung nach Latarjet ist eine häufig angewandte Operationstechnik in allen Altersstufen [5]. Teilweise resultiert eine postoperative Bewegungseinschränkung, jedoch kann in den meisten Fällen eine stabile Gelenkführung und Schmerzlinderung erreicht werden. Bei älteren Patienten, insbesondere an Epilepsie leidende Menschen mit geringem körperlichem Anspruch, stellt auch das Belassen der Situation eine mögliche Therapieoption dar [8]. Benetti et al. [3] schlagen als Voraussetzung für einen konservativen Therapieversuch einen minimalen schmerzfreien Bewegungsumfang von 50° vor, weisen jedoch auch daraufhin, dass eine Schmerzlinderung selten erreicht werden kann.

Chronische vordere Schulterluxationen bei jungen Patienten sollten daher eher offen reponiert werden. Abhängig von der individuellen Anatomie und Begleitverletzungen sollte dann die entsprechende operative Rekonstruktion erfolgen. Durch das hier vorgestellte operative Verfahren konnte bereits im kurzfristigen Follow-up ein zufriedenstellendes Ergebnis erzielt werden. Der eigenen Kenntnis nach ist die Kombination der vorgestellten Techniken bei der chronischen vorderen Schulterluxation bisher noch nicht in der Literatur beschrieben. Ferner zeigt die dargestellte Kasuistik, dass eine verspätete fachärztliche Konsultation zum Übersehen häufiger Verletzungen, wie der ventralen Schulterluxation, führen kann. Vor allem die medizinische Versorgung von Menschen in Pflegeeinrichtungen kann aufgrund von Einschränkungen im Rahmen von Lockdowns aufgrund der COVID-19-Pandemie zu einer verzögerten Behandlung führen [4].

## Fazit für die Praxis

- Verhakte, vordere Schulterluxationen sind eine Seltenheit und sollten insbesondere bei jüngeren Patienten operativ versorgt werden
- Postoperativ stehen die Stabilität und Schmerzfreiheit im Vordergrund.
- Neben der Schulterstabilisierung sollten größere Hill-Sachs-Defekte von mehr als 40 % des Humeruskopfdurchmessers aufgefüllt werden, z. B. mit Autograft des Beckenkamms.
- Eine orthopädische Patientenversorgung sollte auch unter eingeschränkten Bedingungen im Rahmen der COVID-19-Pandemie gewährleistet sein.
- Insbesondere vulnerable Gruppen wie Heimbewohner, die darüber hinaus in der Kommunikation eingeschränkt sind, müssen bei muskuloskeletalen Beschwerden einer fachärztlichen Behandlung zugeführt werden.
